# Risk factors for cardiovascular mortality in patients with systemic lupus erythematosus, a prospective cohort study

**DOI:** 10.1186/ar3759

**Published:** 2012-03-05

**Authors:** Johanna T Gustafsson, Julia F Simard, Iva Gunnarsson, Kerstin Elvin, Ingrid E Lundberg, Lars-Olof Hansson, Anders Larsson, Elisabet Svenungsson

**Affiliations:** 1 Rheumatology Unit, Department of Medicine, Karolinska University Hospital, Solna, Karolinska Institutet, SE-171 76 Stockholm, Sweden; 2 Clinical Epidemiology Unit, Department of Medicine, Karolinska Institutet, SE-171 76 Stockholm, Sweden; 3 Unit of Clinical Immunology, Department of Clinical Immunology and Transfusion Medicine, Karolinska University Hospital, Solna, Karolinska Institutet, SE-171 76 Stockholm, Sweden; 4 Department of Laboratory Medicine, Karolinska University Hospital, Karolinska Institutet, SE-171 76 Stockholm, Sweden; 5 Department of Clinical Chemistry and Pharmacology, Akademiska Hospital, SE-751 85 Uppsala, Sweden

## Abstract

**Introduction:**

Systemic lupus erythematosus (SLE) is a chronic autoimmune disease. Cardiovascular disease (CVD) is common and a major cause of mortality. Studies on cardiovascular morbidity are abundant, whereas mortality studies focusing on cardiovascular outcomes are scarce. The aim of this study was to investigate causes of death and baseline predictors of overall (OM), non-vascular (N-VM), and specifically cardiovascular (CVM) mortality in SLE, and to evaluate systematic coronary risk evaluation (SCORE).

**Methods:**

208 SLE patients were included 1995-1999 and followed up after 12 years. Clinical evaluation, CVD risk factors, and biomarkers were recorded at inclusion. Death certificates and autopsy protocols were collected. Causes of death were divided into CVM (ischemic vascular and general atherosclerotic diseases), N-VM and death due to pulmonary hypertension. Predictors of mortality were investigated using multivariable Cox regression. SCORE and standardized mortality ratio (SMR) were calculated.

**Results:**

During follow-up 42 patients died at mean age of 62 years. SMR 2.4 (CI 1.7-3.0). 48% of deaths were caused by CVM. SCORE underestimated CVM but not to a significant level. Age, high cystatin C levels and established arterial disease were the strongest predictors for all- cause mortality. After adjusting for these in multivariable analyses, only smoking among traditional risk factors, and high soluble vascular cell adhesion molecule-1 (sVCAM-1), high sensitivity C-reactive protein (hsCRP), anti-beta2 glycoprotein-1 (abeta2GP1) and any antiphospholipid antibody (aPL) among biomarkers, remained predictive of CVM.

**Conclusion:**

With the exception of smoking, traditional risk factors do not capture the main underlying risk factors for CVM in SLE. Rather, cystatin C levels, inflammatory and endothelial markers, and antiphospholipid antibodies (aPL) differentiate patients with favorable versus severe cardiovascular prognosis. Our results suggest that these new biomarkers are useful in evaluating the future risk of cardiovascular mortality in SLE patients.

## Introduction

Systemic lupus erythematosus (SLE) is an autoimmune rheumatic disease predominately affecting women (90%). Clinical manifestations are systemic, affecting organs including skin, joints, kidneys and the vascular system.

Cardiovascular disease (CVD) is a well studied co-morbidity of SLE with many remaining questions to be answered. Both subclinical CVD, measured as atherosclerosis, and clinical events have been subjects for investigation. Studies have focused on different aspects of the disease to find associations with, and to characterize, SLE-related CVD. For example, CVD in SLE has been associated with clinical manifestations, disease activity and damage, traditional and non-traditional riskfactors, and demographic factors [1-4]. Risk factors for cardiovascular mortality (CVM*) *in SLE on the other hand, have not yet been well studied.

In the 1950s, the estimated 5-year survival was less than 50% [5], but recent studies report 5-year survival of over 90% [6,7]. Nevertheless, the mortality rate in SLE still exceeds that of the general population [8,9]. Death related to lupus activity and infection has decreased over time, but still contributes to mortality [10,11], especially in developing countries[12,13]. However, CVM has not declined [14] in SLE. A slight increased standardized mortality ratio (SMR) due to vascular diseases has been reported [15], and death from CVD accounts for between 17% and 76% in different studies [16,17]. To date, most studies have investigated risk factors for overall mortality (OM), sometimes with diverging results [10,12,18-21]. As CVM accounts for a growing part of mortality in SLE, it is important to identify risk factors specifically for CVM. In the general population, the systematic coronary risk evaluation (SCORE) [22] is a well-established tool to predict the 10-year risk of CVM based on traditional risk factors. SCORE has not previously been evaluated in SLE. Many new biomarkers that could help identify underlying molecular pathways of importance for vascular damage, such as endothelial and inflammatory markers and cystatin C have not been evaluated with respect to mortality in SLE.

Therefore, we described a large set of biomarkers and SCORE in a cohort of 208 SLE patients from a single center. We determined causes of death and the contribution of baseline predictors for OM, CVM and non-vascular mortality (N-VM).

## Materials and methods

During the inclusion period (1995 to 1998), 208 patients with prevalent disease, who were attending the Department of Rheumatology, Karolinska University Hospital, and fulfilled four or more of the 1982 revised American College of Rheumatology criteria for classification of SLE [23] were included. Most patients (94%) were European Caucasians. The Local Ethics Committee at Karolinska University Hospital approved the study and patients provided informed consent.

At inclusion, all data were collected in one session for each patient. A rheumatologist interviewed and examined patients according to a structured protocol. Medical history, traditional CVD risk factors (smoking, hypertension, hypercholesterolemia, diabetes) and medication were reviewed, through interviewing the patient and by studying medical records. SLE disease activity was determined using the Systemic Lupus Activity Measure (SLAM) [24] and organ damage was assessed using the Systemic Lupus International Collaborating Clinics (SLICC) damage index [25]. Laboratory examinations were performed on fasting fresh blood samples or on samples stored at -70°C. When stored samples were used, each analyte was assayed in one session.

Survival status was followed up in the national population registry on March 26, 2010, after a mean time of 12.3 years. Two patients were interviewed by telephone as they had moved abroad. Death certificates were collected from the Cause of Death Register of The National Board of Health and Welfare. When available, autopsy protocols were collected from the department of Pathology, Karolinska University Hospital (n = 10), and from the Department of Forensic Medicine (n = 4). Causes of death were based on information from death certificates, autopsy protocols and medical records. Two clinicians (JG and ES) classified all causes of death together as follows: CVM (death due to myocardial infarction, atherosclerosis, heart failure, ischemic cerebrovascular disease or sudden death), death due to pulmonary hypertension (PHT) and N-VM.

### Laboratory methods

High sensitivity C-reactive protein (hsCRP), α-1 antitrypsin, fibrinogen and serum amyloid A (SAA) were measured using BN ProSpec System (Dade Behring, Göttingen, Germany). Complement factors C3 and C4 were analyzed using IMMAGE™ and C3d using an ARRAY™ system (both instruments from Beckman Coulter, Brea, CA, USA). Apolipoprotein A1, apolipoprotein B, creatinine, high-density lipoprotein (HDL), low-density lipoprotein (LDL) and total cholesterol, triglycerides and urea were analyzed using the LX20 chemistry analyzer (Beckman Coulter, Brea, CA, USA). Homocysteine was assayed using the IMx system and Cystatin C was analyzed using the Architect Ci8200 analyzer (both by Abbott Laboratories, Chicago IL, USA). ELISA was used to measure soluble vascular cell adhesion molecule (sVCAM-1) and IL-6 (DY809 and HS600, R&D Systems, Minneapolis, MN, USA) and von Willebrand factor (vWF) (antisera from Dako, Glostrup, Denmark) was calibrated against Liatest (Diagnostica Stago, Asnières sur Seine, France). The intra-assay coefficient of variation for the ELISAs was < 7%.

Antinuclear antibodies (ANA) was analyzed by immunofluorescence on HEp-2 cells (Immunoconcepts, Sacramento, CA, USA) and antibodies to Sjogrens syndrome A and B (SSA, SSB), Smith antigen (Sm) and Ribonucleoprotein (RNP) using ANA-profile ELISA (Thermo Fisher Scientific, previously Phadia, Uppsala, Sweden), Innolia immunoblot (Innogenetics, N.U. Ghent, Belgium), and immunodiffusion (Immunoconcepts, Sacramento, CA, USA). Anti-dsDNA was determined by *Crithidia lucillae *kinetoplast assay (Immunoconcepts, Sacramento, CA, USA). Anticardiolipin antibodies (aCL) were measured by ELISA using ethanol-fixed cardiolipin (Sigma-Aldrich, St Louis, MO, USA) and horseradish peroxidase (HRP)-conjugated rabbit anti-human IgG and IgM (Dako, Glostrup, Denmark). Positivity was calibrated against Harris standard (Louisville, LAPL-GM-001, Louisville, KY, USA) [26]. Low aCL level corresponded to 10 to 20, medium level to 20 to 80 and high level to > 80 GPU/mPL units. The cutoff values for positive aCL was calculated to be at least the 95^th ^percentile of healthy blood donors. Autoantibodies to β2-glycoprotein1 (aβ_2_GP1, IgG) were analyzed by ELISA (Orgentec Diagnostika, Mainz, Germany). Positive cutoff levels were used according to the manufacturers' descriptions. Borderline results were regarded as negative. Lupus anticoagulant (LAC) was determined using a modified dilute Russell viper venom method, (Biopool, Umea, Sweden) using Bioclot LAC.

### Statistics

Patient characteristics were summarized overall and stratified by outcome using analysis of variance (ANOVA), Mann-Whitney *U*-test and Chi^2 ^test, as appropriate. Skewed continuous variables were log transformed before use in parametric analyses. The SMR for all deaths and corresponding 95% confidence interval (CI) assuming a Poisson distribution was calculated using age-, sex-, and calendar year-specific mortality rates for the Swedish population to estimate the expected number of deaths. Hazard ratios (HRs), it should be hazard ratios everywhere and 95% CI for OM, CVM and N-VM were calculated in age-adjusted Cox models for baseline factors. The proportional hazards assumption was evaluated by assessing the significance of the interaction between predictors and follow-up time and the assumption was met. PHT was included in OM but otherwise excluded because of too few cases. The limited number of deaths restricted the variables in the multivariable-adjusted model to four. The two predictors most strongly associated with mortality after age adjustment (in terms of *P*-value) in the three groups (OM, CVM, N-VM) were retained in all multivariable analyses. Thereafter, each baseline variable was considered separately in multivariable models.

To evaluate the different multivariable models predicting CVM in terms of identifying the best model, and to compare that model with SCORE, Akaike information criterion (AIC) values were compared using logistic regression. To consider the possibility of effect modification by sex, we stratified by sex; we restricted the sensitivity analysis to the female subset. Male patients were not considered due to small sample size. Possible effect modification on cystatin C by steroid treatment was evaluated by stratifying by steroid treatment. To account for the possibility that subclinical or unregistered nephritis could affect the cystatin C results, we also stratified by history of nephritis.

SCORE [22] was calculated using the Swedish Heartscore. Baseline data on age, smoking, sex, systolic blood pressure and cholesterol were incorporated into the web-based formula [27]. The 10-year risk of CVM was calculated for patients between 40 to 65 years of age, according to the SCORE protocol. The estimated number of CVMs was compared to the observed number using Fisher´s exact analysis.

Multiple imputation was used for missing data. For dichotomous variables the models were re-run assuming each possible value and the results were compared. For continuous predictors, three imputed values were used: the minimum, the mean, and the maximum value. Descriptive statistics and regression analyses were done using JMP software (SAS Institute, Carey, North Carolina, USA) and SAS 9.2 was used for SMR calculations. A *P*-value ≤ 0.05 was considered statistically significant.

## Results

All patients were followed up. Forty-two patients (20%) died, 36 women and 6 men, at a mean age of 62 years (women 61 ± SD 14, men 64 ± 19 years). More deaths were observed than expected (SMR = 2.4, 95% CI 1.7 to 3.0). CVM was the predominant cause of death (n = 20, 48%) (Table 1).

**Table 1:** Causes of death in deceased patients (n = 42)

Primary cause of death	Number of patients	Age at death, years	Age at SLE diagnosis, years	Disease duration at baseline, years
**Cardiovascular mortality**	**20 (48%)**	**69 (± 11)**	**43 (± 18)**	**19 (± 14)**

Myocardial infarction	10			
Congestive.heart failure	7			
CVLcerebrovascular lesion	1			
Atherosclerosis	1			
Sudden death	1			

**Non-vascular mortality**	**18**	**57 (± 13)**	**36 (± 13)**	**15 (± 10)**

Infection	5 (12%)	46 (± 12)	20 (± 6.8)	16 (± 10)
Sepsis	3			
Pneumonia	1			
Perforation of esophagus	1			
Bleeding	4 (10%)	56 (± 16)	32 (± 13)	20 (± 9)
Gastrointestinal bleeding	3			
Unknown	1			
Malignancy	4 (10%)	62 (± 6)	37 (± 10)	18 (± 9)
Lung cancer	2			
Colorectal cancer	1			
Squamous cell cancer	1			
Suicide	2 (5%)	64 (± 3)	51 (± 19)	11 (± 13)
Pulmonary disease (pneumothorax)	1 (2%)	54	46	6
Renal failure	1 (2%)	85	73	2
Hepatic failure (cirrhosis)	1 (2%)	67	54	2

**Pulmonary hypertension**	**4 (10%)**	**41 (± 16)**	**29 (± 13)**	**10 (± 9)**

**Total/Average**	**42**	**62 (± 14)**	**33 (± 13)**	**13 (± 10)**

Age is given as mean ± SD.

At inclusion, 124 patients were between 40 and 65 years of age. In this group, we observed nine cardiovascular deaths within 10 years. SCORE only predicted four deaths and the difference was not statistically significant (odds ratio, OR 2.3, 95% CI 0.7 to 7.8, *P *= 0.25).

Several parameters measured at baseline differed between deceased and surviving patients (Table 2) and persisted after age adjustment for all outcomes. Established arterial disease and Cystatin C were the strongest risk factors in all groups (Table 3) and were retained in the remaining analyses (Table 4). OM was predicted by several inflammatory parameters, sVCAM-1 and SLICC >1.

**Table 2:** Baseline characteristics of patients with SLE (n = 208)

	All patients n = 208	Deceased patients n = 42	Surviving patients n = 166	*P*
**Traditional risk factors**				
Age	47 (35-54)	57 (48-65)	45 (31-52)	< 0.0001
Male gender	11%	14%	10%	
Smoking	24%	26%	22%	
Hypertension	34%	62%	28%	< 0.0001
Systolic blood pressure, mmHg	125 (115-140)	140 (123-148)	120 (110-140)	0.001
Hypercholesterolemia	44%	55%	41%	
Total cholesterol, (mmol/l)	5.1 (4.3-6.0)	5.6 (4.7-6.7)	5.0 (4.3-5.9)	0.04
Low density lipoprotein mmol/l	2.9 (2.3-3.6)	3.3 (2.2-3.9)	2.8 (2.3-3.6)	
High density lipoprotein, mmol/l	1.4 (1.1-1.7)	1.4 (1.1-1.9)	1.4 (1.1-1.7)	
Triglycerides, mmol/l*	1.3 (1.0-2.0)	1.7 (1.2-2.5)	1.2 (0.9-1.9)	0.004
ApolipoproteinB/ApolipoproteinA*#	0.5 (0.3-0.6)	0.5 (0.3-0.8)	0.5 (0.3-0.6)	
Diabetes	3%	10%	2%	0.03
Established arterial disease	13%	40%	5%	<0.0001
SCORE	3.2% (± 2.8)	4.4% (± 2.4)	2.7% (± 2.4)	0.005
**Lupus manifestations **(ever present)§				
Age at disease onset, years	30 (22-40)	35 (25-52)	28 (21-40)	0.0002
Disease duration, years	12 (5-20)	13.5 (6-26)	12 (5-19)	0.008
Malar rash	56%	50%	58%	
Discoid rash	20%	12%	21%	
Photosensitivity	71%	60%	73%	
Oral ulcers	28%	28%	26%	
Arthritis	86%	86%	86%	
Pleuritis	40%	52%	37%	
Pericarditis	18%	17%	19%	
Nephritis	35%	40%	34%	
Neurological disorder	15%	29%	12%	0.009
Leucopenia	51%	37%	55%	0.04
Thrombocytopenia	22%	30%	20%	
Previous venous occlusion	11%	17%	9%	
SLICC > 1 [25]	59%	93%	50%	< 0.0001
SLAM > 6 [24]	59%	73%	55%	0.03
**Autoantibodies against** **(at baseline if not stated otherwise)**				
Double-stranded DNA	38%	43%	36%	
Double-stranded DNA (ever)	61%	64%	60%	
Cardiolipin IgG low titer	48%	50%	47%	
Cardiolipin IgG medium titer	18%	31%	15%	0.02
Cardiolipin IgM low titer	16%	19%	15%	
Cardiolipin IgM medium titer	8%	14%	6%	
beta_2_glykoprotein-1#	22%	31%	20%	
Lupus anticoagulant	23%	31%	21%	
Any antiphospholipid low titer¶	60%	62%	60%	
Any antiphospholipid medium titer¶	39%	55%	36%	0.03
Sjogrens syndrome A	41%	21%	46%	0.003
Sjogrens syndrome B	22%	10%	25%	
Smith	10%	7%	10%	
Ribonucleoprotein	19%	21%	19%	
**Medication**				
Steroids	51%	48%	62%	
Cyclofosfamid (ever use)	17%	26%	16%	
Azathioprine	12%	12%	12%	
Chloroquine/hydroxychloroquine	27%	14%	30%	0.04
Warfarin	13%	29%	10%	0.001
Acetylsalicylic acid	21%	33%	18%	0.02
Statins	2%	5%	1%	0.05
Methotrexate	4%	7%	1%	
Cyclosporine	3%	10%	1%	0.004
**Inflammatory markers**				
High sensitivity C reactive protein, mg/l* #	2.2 (0.8-6.2)	5.1 (2.2-12.1)	1.8 (0.7-4.5)	0.0002
Fibrinogen, g/l* #	3.7 (2.9-4.6)	4.3 (3.5-5.2)	3.4 (2.9-4.4)	< 0.0001
α-1 antitrypsine, g/l #	1.5 (1.3-1.8)	1.7 (1.5-2.1)	1.5 (1.3-1.7)	0.0001
Serum amyloid A, mg/l* #	5.4 (2.7-12)	10.5 (5.9-21.6)	4.7 (2.4-9.6)	
Interleukin-6, ng/l* #	3.5 (2.1-7.0)	5.2 (2.9-9.7)	3.1 (2.0-6.2)	
Complement factor 3, g/l #	1.0 ( 0.8-1.2)	1.0 (0.7-1.3)	1.0 (0.8-1.2)	
Complement factor 3 degradation products, mg/l* #	11.9 (9.6-14.9)	13.4 (10.8-16.6)	11.6 (9.5-14.5)	0.02
Complement factor 4 (g/l)* #	0.2 (0.1-0.2)	0.2 (0.1-0.2)	0.2 (0.1-0.2)	
**Endothelial markers**				
Soluble vascular cell adhesion molecule 1, ng/l* #	315 (258-392)	377 (297-526)	306 (250-375)	< 0.0001
Von Willebrand factor, %*#	120 (63-175)	144 (102-231)	116 (60-164)	0.003
**Markers of renal damage**				
Creatinine	82 (73-95)	91 (75-138)	81 (73-92)	< 0.0001
Modification of Diet in Renal Disease formula, ml/min/1.73m2	66.6 (54.3-79.1)	54.6 (36.6-76.9)	67.7 (59.5-79.9)	0.0005
Cystatin C GFR	80 (61-107)	55 (27-83)	86 (71-114)	< 0.0001
Cystatin C, mg/l*	1.0 (0.8-1.2)	1.3 (1.0-2.2)	0.9 (0.8-1.1)	< 0.0001
Blod urea nitrogen, mmol/l* #	5.8 (4.7-7.3)	7.3 (6.1-13.6)	5.5 (4.5-6.7)	
Pathologic urine	24%	22%	29%	
**Other biomarkers**				
Homocysteine, mol/l* #	12.5 (10.1-16.5)	14.3 (10.8-20.6)	12 (10.0-15.3)	0.002

Distributions are given as % or median (interquartile range), except for SCORE, where mean values are given. *P*-values ≤ 0.05 are presented. *Variables with non-normal distribution. # Analysis of frozen samples. §According to American College of Rheumatology (ACR) criteria [23]. ¶Positive antibody against cardiolipin IgG/IgM at low and medium titer, respectively, beta_2_glykoprotein-1, or a positive lupus anticoagulant test. Hypertension, systolic blood pressure > 140 mm Hg and/or diastolic blood pressure > 90 mmHg and/or treatment for hypertension; hypercholesterolemia, total cholesterol level > 5.2 mmol/L; established arterial disease, history of myocardial infarction, angina pectoris, ischemic cerebrovascular disease, ischemic peripheral arterial disease at baseline; SCORE, systematic coronary risk evaluation calculated on 124 patients 40 to 65 years old; SLICC, systemic lupus international collaborating clinics; pathologic urine, as defined by the systemic lupus activity measure (SLAM). Data were missing as follows (deceased/survivors): low and high density lipoprotein (5/11), triglycerides (0/1), Apolipoprotein B/Apolipoprotein A (1/0), leucopenia (1/0), thrombocytopenia (1/3), cyclophosphamide ever (15/3), statins (0/3), cyklosporin (0/1), α-1 antitrypsine (0/1), serum amyloid (1/4), complement factor (C)3 (0/3), C3 degradation products (0/3), C 4 (0/3), soluble vascular cell adhesion molecule 1 (1/2), von Willebrand factor (1/2), cystatin C/cystatin C glomerular filtration rate (GFR) (1/0), urea (3/3), homocysteine (2/5).

**Table 3:** Age-adjusted Cox regression analysis

	OM n = 42		CVM n = 20		N-VM n = 18	
	*P*-value	HR (95% CI)	*P*-value	HR (95% CI)	*P*-value	HR (95% CI)
Age univariate	< 0.0001	1.1 (1.0-1.1)	< 0.0001	1.07 (1.04-1.1)	0.02	1,04 (1.00-1.1)
Age adjusted						
**Traditional risk factors**						

Smoking			0.02	3.3 (1.3-8.6)		
Hypertension	0.02	2.2 (1.1-4.5)			0.04	2.8 (1.0-7.9)
Cholesterol, mmol/l						
Triglycerides, mmol/l†	0.02	1.9 (1.1-3.3)			0.04	2.5 (1.1-5.7)
Established arterial disease	< 0.0001	4.7 (2.4-9.1)	0.0006	5.4 (2.1-13.6)	0.0006	6.3 (2.3-16.9)
**Lupus manifestations**						

Nephritis			0.03	3.0 (1.2-7.9)		
Neurological disorder			0.04	2.9 (1.1-7.3)		
Epilepsy	0.03	2.3 (1.1-4.5)	0.01	3.7 (1.4-9.3)		
SLICC > 1 [25]	<0.0001	6.3 (2.2-26.5)	0.04	3.7 (1.0-23.5)	0.002	9.6 (1.9-181.1)
SLAM > 6 [24]	0.02	2.1 (1.1-4.5)				
**Autoantibodies against**						

Double-stranded DNA	0.05	1.9 (1.0-3.6)			0.02	3.1 (1.2-8.3)
Cardiolipin IgG low titer					0.03	2.9 (1.1-8.1)
Cardiolipin IgG medium titer	0.006	2.7 (1.4-5.3)	0.04	3.2 (1.1-8.5)	0.02	3.6 (1.3-9.6)
beta_2_glykoprotein-1			0.04	2.9 (1.0-7.8)		
Any aPL medium titer ¶	0.02	2.1 (1.1-3.9)	0.03	2.8 (1.1-7.7)	0.03	2.8 (1.1-7.6)
Sjogrens syndrome A	0.01	0.4 (0.2-0.8)				
Sjogrens syndrome B	0.04	0.4 (0.1-0.9)			0.003	5 × 10^-7^ (0-0.4)
**Medications**						

Warfarin	0.004	2.9 (1.4-5.7)	0.01	4.1 (1.4-11.1)	0.03	3.3 (1.1-8.3)
Hyperlipidemia			0.03	7.8 (1.2-27.9)		
**Inflammatory markers**						

High sensitivity CRP, mg/l†	0.01	1.3 (1.0-1.7)	0.02	1.5 (1.1-2.1)		
Fibrinogen, g/l†	0.0002	9.5 (3.0-31)	0.02	10.2 (1.6-70)	0.0005	19.9 (3.8-110)
α-1 antitrypsin, g/l	0.002	3.1 (1.5-5.8)			0.001	5.1 (2.0-12.0)
Serum amyloid A, mg/l†	0.01	1.4 (1.1-1.8)	0.03	1.5 (1.0-2.1)		
Endothelial markers						
Soluble vascular cell adhesion molecule, ng/l†	0.0005	4.0 (1.8-8.2)	0.004	4.9 (1.7-13.5)		
Von Willebrand factor, %†	0.009	1.9 (1.2-3.0)				
**Markers of renal damage**						

Cystatin C GFR	< 0.0001	0.4 (0.2-0.5)	0.001	0.3 (0.2-0.6)	0.0002	0.3 (0.2-0.5)
Cystatin C†	< 0.0001	4.4 (2.4-7.9)	0.0009	5.3 (2.0-13)	0.0002	5.7 (2.4-12.8)
Creatinine	0.003	1.1 (1.0-1.1)	0.02	4.1 (1.3-10.2)	0.003	4.3 (1.7-9.2)
Urea, mmol/l†	0.01	2.1 (1.2-3.2)			0.02	2.3 (1.1-4.0)
Pathologic urine	0.04	2.2 (1.0-4.4)			0.04	3.5 (1.1-8.1)

Only variables with *P*-values ≤ 0.05 in either group are presented. Calculations were done using age-adjusted Cox regression. †Calculations on log transformed values. Any aPL¶, any antiphospholipid antibody, positive antibody against cardiolipin IgG/IgM at medium titer, beta_2_glykoprotein-1 or a positive lupus anticoagulant test. Hypertension, systolic blood pressure > 140 mm Hg and/or diastolic blood pressure > 90 mmHg and/or treatment for hypertension; hypercholesterolemia, total cholesterol level > 5.2 mmol/L; established arterial disease, history of myocardial infarction, angina pectoris, ischemic cerebrovascular disease, ischemic peripheral arterial disease at baseline; SCORE, systematic coronary risk evaluation calculated on 124 patients 40 to 65 years old; SLICC, systemic lupus international collaborating clinics; pathologic urine, as defined by the systemic lupus activity measure (SLAM); OM, overall mortality; CVM, cardiovascular mortality; N-VM, non-vascular mortality. HR, hazard ratio

**Table 4:** Multivariable Cox regression model adjusted for age, arterial disease and cystatin C (208 patients).

	OM		CVM		N-VM	
	*P*-value	HR (95% CI)	*P*-value	HR (95% CI)	*P*-value	HR (95% CI)
Smoking			0.02	3.4 (1.3-9.2)		
SLICC>1[25]	0.008	4.1 (1.4-17.3)			0.05	5.6 (1.0-103.6)
aβ_2_GP1			0.03	3.4 (1.2-9.7)		
Any aPL medium titer			0.05	2.8 (1.0-8.2)		
Sjogrens syndrome B antibodies					0.02	1.3e-6 (0-0.7)
Warfarin			0.05	3.4 (1.0-10.4)		
High sensitivity CRP	0.04	1.3 (1.0-1.6)	0.02	1.6 (1.1-2.3)		
Fibrinogen	0.04	3.7 (1.0-13.1)			0.05	6.7 (1.0-45.4)
α-1-antitrypsine	0.007	2.7 (1.3-5.2)			0.004	4.3 (1.6-10.7)
Soluble vascular cell adhesion molecule 1	0.05	2.7 (1.0-6.7)	0.02	5.3 (1.3-19.3)		

aβ_2_GP1, anti-β_2 _glycoprotein-1; any aPL, any antiphospholipid; positive antibody against cardiolipin IgG/IgM at medium titer, β_2_GP1 or a positive lupus anticoagulant test; OM, overall mortality; CVM, cardiovascular mortality; HR, hazard ratio; N-VM, non-vascular mortality; SLICC, systemic lupus international collaborating clinics. Age, arterial disease and cystatin C were included in all multivariable models. Each variable that was significant in age-adjusted Cox regression (Table 3) for OM, CVM and N-VM, was included one by one, together with the three above-mentioned variables, creating a model with four variables. The table presents the results of the significant associations. Only variables that have *P*-values ≤ 0.05 in either group are presented. In this way, five different multivariable models are presented for OM, six for CVM, and four for N-VM, respectively.

Smoking was the only traditional risk factor predicting CVM. sVCAM-1, hsCRP, aβ_2_GP1, any aPL at medium titer, and baseline warfarin treatment also predicted CVM. N-VM was positively associated with markers of systemic inflammation and SLICC >1, while SSB autoantibodies were inversely associated (Table 4). Results were similar among women, with a few exceptions (see Table 5). The best multivariable model predicting CVM included age, established arterial disease, cystatin C and smoking (AICc value 77). Yes, the same thing All six multivariable models predicting CVM (AICc values ranging from 77 to 85) performed better than SCORE (AICc value 122).

**Table 5:** Multivariable Cox regression model adjusted for age, arterial disease and cystatin C

	OM		CVM		N-VM	
	*P*-value	HR (95% CI)	*P*-value	HR (95% CI)	*P*-value	HR (95% CI)
Smoking			0.02	3.6 (1.3-10.4)		
SLICC >1 [25]	0.01	3.8 (1.3-16.4)				
aβ_2_GP1			0.02	3.6 (1.2-10.6)		
Sjogrens syndrome B antibodies					0.02	9.1e-7 (0-0.8)
Warfarin			0.04	3.7 (1.1-11.9)		
High sensitivity CRP			0.01	1.6 (1.1-2.4)		
Fibrinogen						
α-1-antitrypsine	0.02	3.0 (1.4-6.4)	0.04	3.3 (1.1-9.2)	0.02	4.3 (1.4-12.7)
Soluble vascular cell adhesion molecule 1			0.02	5.2 (1.3-18.8)		

aβ_2_GP1, anti-β_2 _glycoprotein-1; SLICC, systemic lupus international collaborating clinics; CRP, C-reactive protein; HR, hazard ratio; OM, overall mortality; CVM, cardiovascular mortality; N-VM, non-vascular mortality. Only *P*-values ≤ 0.05 are presented.

Stratification by steroid treatment did not influence the impact of cystatin C on mortality. Among those without history of nephritis (n = 135 of whom 25 died cystatin C adjusted for age remained significant; *P *= 0.009, RR 4.6 (95% CI 1.5 to 14.5). For patients with history of nephritis (n = 73 of whom 17 died), the results for cystatin C were similar; *P *= 0.001, RR 4.4 (95% CI 1.8 to 10.7).

Results were comparable under the numerous imputed scenarios with the exception of cyclophosphamide, where a large proportion of missingness was observed among deceased patients, and alternative imputed scenarios yielded different results. As these were considered unreliable, they were removed from consideration.

## Discussion

To our knowledge, this is the first study to prospectively examine risk factors specifically for CVM, which accounted for almost 50% of deaths in our cohort. Additionally, 10% of patients died from PHT. CVM and N-VM shared many risk factors. Established arterial disease, Cystatin C and inflammatory markers were strong predictors for both, but s-VCAM-1, a marker of endothelial cell activation, was only associated with CVM. Also, aβ_2_GP1 and any aPL at medium titer predicted CVM. SCORE underestimated the risk for CVM, but the results were not statistically significant. Consistent with recently published work [9,15], SMR was 2.4. Our survival rate of 80% after a mean follow-up of approximately 12 years is generally consistent with previous findings, which range from 76% to 92% survival after 10 years [28,29]. Our results confirm [6,17,30] that composite damage (SLICC > 1[25]) predicted OM, but our focus was to analyze the impact of different organ manifestations and immunological profile on mortality.

High levels of cystatin C and low estimated glomerular filtration rate (eGFR) based on cystatin C [31] emerged as strong predictors for all outcomes. These associations were independent of inflammatory and endothelial biomarkers and were not modified by steroid treatment at baseline. Creatinine and eGRF, calculated using the Modification of Diet in Renal Disease (MDRD) formula [32] did not predict mortality.

Renal disease in lupus is associated with poor prognosis [10,20,33]. Cystatin C has been proposed as a more reliable biomarker for renal function than creatinine as it rises with smaller reductions in GFR [34], and is less influenced by age, sex, muscle mass and diet [35]. Nevertheless, cystatin C levels may be affected by glucocorticoid use [36] and inflammation [37], both often present in SLE. Cystatin C has furthermore emerged as a marker of CVD risk [38], CVM and N-VM in subjects with normal eGFR [39]. The fact that the nephritis manifestation in our study only influenced CVM to a modest degree, while cystatin C predicted mortality significantly both in patients with and without reported history of nephritis, further emphasizes the importance of cystatin C as a new useful biomarker. Our results demonstrate that cystatin C is strongly predictive of mortality and that it merits further evaluation as a biomarker in SLE.

sVCAM-1 was a strong risk factor for OM and in particular for CVM, underscoring the importance of endothelial activation for CVM in lupus. Endothelial biomarkers have not previously been investigated in the context of mortality in SLE. Levels of sVCAM-1 are elevated in SLE patients with manifest CVD [40] and together with vWf, another endothelial marker, they predicted the first arterial event [26]. An association with atherosclerosis has been demonstrated both in the general population and in lupus [4].

Systemic inflammation is associated with CVD, CVM and N-VM in the general population [41,42] and may be associated with CVD in lupus [26,43-45], although the impact on mortality has not yet been well studied. We demonstrated that several inflammatory markers were associated with all-cause mortality. For CVM, hsCRP (and α1-antitrypsin among women) had the greatest impact.

aβ_2_GP1 and any aPL at medium titer were associated with CVM in multivariable analyses. This observation is in accordance with previous studies, where aPLs were associated with cardiovascular events [26,44]. The high prevalence of aCL in our study is probably due to a low cutoff for positivity at our laboratory in the mid 90's, when baseline data were collected. Since then, much work has been carried out to evaluate more appropriate cutoffs for aCL in relation to what is clinically significant [46]. When we used a cutoff at medium titer, the prevalence of aCL was more in accordance with other studies [47]. Furthermore, medium titer of aPLs were predictive of CVM, indicating that these are more clinically relevant levels. However, we have previously demonstrated that aPL only at a low cutoff were predictive of the first arterial event [26]. aβ_2_GP1 was analyzed later on frozen samples, with the method currently used at our immunological laboratory. Taken together, our results demonstrate that clinically relevant levels for aPL need to be further studied and standardized. Studies in our research group are ongoing to investigate these issues.

SSA/SSB antibodies often occur together with skin manifestations in SLE. We noted an inverse association between positivity for SSB antibodies and N-VM. SSA antibodies [20] and photosensitivity [10] were previously inversely linked to mortality. Together these results indicate that lupus patients with SSA/SSB positivity and/or skin manifestations have a better prognosis, further illustrating that sub-phenotypes of SLE have differentiated risk profiles [26].

Smoking was the only traditional CVD risk factor associated with CVM in multivariable analyses. Smoking has previously been shown to predict cardiovascular events in SLE [26,44]. Hypertension was predictive of mortality in earlier SLE studies [19,48]. As definitions differ and antihypertensive agents are used in the treatment of nephritis, even in the absence of high blood pressure, it is nowadays difficult to assess its contribution to mortality. Hyperlipidemia was not an important risk factor for mortality in multivariable analyses.

SCORE is a widespread clinical cardiovascular risk scoring system distributed through the European Society of Cardiology. SCORE underestimated CVM among our SLE patients (nine observed vs. four predicted cases), but the difference was not significant. This is nevertheless interesting as it suggests that optimal preventive cardiovascular strategies in lupus need to target other factors in addition to traditional CVD risk factors.

Notably, four SLE patients (10%) in this cohort died from PHT. They died at a young mean age (41 years). Death from PHT was 15% in a Korean cohort [49], but in most studies PHT is not reported as a prominent cause of death.

Detailed baseline information and complete follow-up are strengths of this study. Assigning a principle cause of death is difficult, particularly in patients with chronic diseases with numerous co-morbidities. We did not rely on death certificates only, but supplemented these data with autopsy protocols and medical charts, thus using all available sources to determine a main cause of death. Because national mortality data are based solely on international classification of diseases (ICD) codes, derived from only one of the data sources we considered in the determination of cause of death, cause-specific SMRs could not be calculated. It is difficult to compare causes of death in our cohort and in the general population for the same reason.

The majority of our patients were female, and of European Caucasian origin. Because Swedish healthcare is tax-funded, granting universal access, patients with lower socioeconomic status have the same access with the same threshold maximum payment per year. Therefore further studies in male patients, other ethnic cohorts and socioeconomic groups are needed. Furthermore, we had limited statistical power. For example, only 13 deaths occurred among patients aged 50 years or younger, prohibiting the evaluation of effect modification by age. Another limitation of this study is the assessment of risk factors at baseline only, which makes it difficult to evaluate development of disease manifestations and other events during follow-up. Finally, we did not adjust for multiple comparisons, and therefore *P*-values close to 0.05 should be interpreted with caution.

## Conclusions

This study demonstrates that high levels of cystatin C strongly predicted all cause mortality. Additionally, CVM was associated with high levels of sVCAM-1, hsCRP and aPL, demonstrating that systemic and vascular inflammation, and prothrombotic autoantibodies are important risk factors for CVM. With the exception of smoking, traditional risk factors had less impact. Thus, new biomarkers differentiate SLE patients with favorable vs. more severe prognosis.

## Abbreviations

aβ_2_GP1: anti-β_2 _glycoprotein-1; aCL: anticardiolipin antibodies; AIC: akaike information criterion; ANA: antinuclear antibodies; ANOVA: analysis of variance; aPL: antiphospholipid antibody; CVD: cardiovascular disease; CVM: cardiovascular mortality; eGFR: estimated glomerular filtration rate; ELISA: enzyme-linked immunosorbent assays; HDL: high-density lipoprotein; HRP: horseradish peroxidase; hsCRP: high sensitivity C-reactive protein; IL-6: interleukin 6; LAC: lupus anticoagulant; LDL: low-density lipoprotein; MDRD: modification of diet in renal disease formula; N-VM: non-vascular mortality; OM: overall mortality; PHT: pulmonary hypertension; RNP: ribonucleoprotein; SAA: serum amyloid A; SCORE: systematic coronary risk evaluation; SLAM: systemic lupus activity measure; SLE: systemic lupus erythematosus; SLICC: systemic lupus international collaborating clinics; Sm: smith antigen; SMR: standardized mortality ratio; SSA/SSB: sjogrens syndrome A/B; sVCAM-1: soluble vascular cell adhesion molecule-1; vWf: von willebrand factor.

## Competing interests

The authors declare that they have no competing interests.

## Authors' contributions

JG acquired, analyzed and interpreted the data and drafted the manuscript. JFS analyzed the data and drafted the manuscript. KE coordinated and acquired the analysis of autoantibodies. IEL acquired the data. LOH and AL coordinated and acquired the laboratory data. ES conceived and designed the study, acquired, analyzed and interpreted the data and drafted the manuscript. All authors reviewed and approved the final manuscript.
